# Cuticle deposition duration in the uterus is correlated with eggshell cuticle quality in White Leghorn laying hens

**DOI:** 10.1038/s41598-021-01718-0

**Published:** 2021-11-11

**Authors:** Xia Chen, Zhaoxiang He, Xingzheng Li, Jianlou Song, Mingyi Huang, Xuefeng Shi, Xianyu Li, Junying Li, Guiyun Xu, Jiangxia Zheng

**Affiliations:** 1 National Engineering Laboratory for Animal Breeding and MOA Key Laboratory of Animal Genetics and Breeding, College of Animal Science and Technology, China Agricultural University, Beijing, 100193 China; 2 Institute of Animal Husbandry and Veterinary Medicine, Beijing Academy of Agriculture and Forestry Sciences, Beijing, 100097 China; 3 Shenzhen Agricultural Genome Research Institute, Chinese Academy of Agriculture Sciences, Shenzhen, 440307 China

**Keywords:** Animal breeding, Animal physiology

## Abstract

The cuticle formed in the uterus is the outermost layer as the first defense line of eggshell against microbial invasions in most avian species, and analyzing its genetic regulation and influencing factors are of great importance to egg biosecurity in poultry production worldwide. The current study compared the uterine transcriptome and proteome of laying hens producing eggs with good and poor cuticle quality (GC and PC, the top and tail of the cuticle quality distribution), and identified several genes involved with eggshell cuticle quality (ESCQ). Overall, transcriptomic analysis identified 53 differentially expressed genes (DEGs) between PC versus GC group hens, among which 25 were up-regulated and 28 were down-regulated. No differences were found in the uterine proteome. Several DEGs, including *PTGDS*, *PLCG2*, *ADM* and *PRLR* related to uterine functions and reproductive hormones, were validated by qPCR analysis. Egg quality measurements between GC and PC hens showed GC hens had longer laying interval between two consecutive ovipositions (25.64 ± 1.23 vs 24.94 ± 1.12 h) and thicker eggshell thickness (352.01 ± 23.04 vs 316.20 ± 30.58 μm) (*P* < 0.05). Apart from eggshell traits, other egg quality traits didn’t differ. The result demonstrated eggshell and cuticle deposition duration in the uterus is one of the major factors affecting ESCQ in laying hens. *PTGDS*, *PLCG2*, *ADM* and *PRLR* genes were discovered and might play crucial roles in cuticle deposition by regulating the uterine muscular activities and secretion function. The findings in the present study provide new insights into the genetic regulation of cuticle deposition in laying hens and establish a foundation for further investigations.

## Introduction

The annual *Global Report on Food Crises 2020* by the Food Security Information Network (FSIN) indicates that the world is facing an unprecedented food crisis^1^. As a source of low-cost and high-quality animal protein, poultry production not only meets the basic nutritional needs of humans, but also is an important contributor to the economies and cultures, and thus the demand for poultry egg and meat is increasing^2^. Egg safety is absolutely pivotal to the success of today’s poultry industry, however, egg contamination with pathogenic bacteria is considered one of the leading causes of economic loss in the poultry industry worldwide and represents a threat to public health^3,4^. Furthermore, faced with current environmental pressures and animal welfare requirements, the modern poultry industry, which relies on the extensive use of artificial incubation, is more dependent on strict biosecurity of the egg^5^.

Avian eggshells have evolved multiple physical and chemical barriers in response to microbial challenges, and these barriers are essential for the successful reproduction of avian species as well as to maintain safe and nutritious table eggs for human consumption^6,7^. The eggshell is a complex with several highly ordered and distinct layers (i.e., mammillae, palisades, vertical crystal layer, and cuticle) and the cuticle is the outermost layer as the first defense line of the eggshell against microbial invasions in most avian species^8–10^. The cuticle has been a relatively neglected structure while recent researches on the properties, composition, functions, physiology and genetics of the cuticle have highlighted its important role in ensuring egg biosecurity and quality in poultry production. The cuticle is deposited during the final hour of egg formation in the uterus (shell gland pouch), covering the shell surface and filling the external openings of the gas pores so that effectively exerting waterproof and antibacterial effects^11,12^. The cuticle mainly consists of hydroxyapatite crystals, glycoprotein, polysaccharides, lipids, and pigment^13,14^, and abundant antibacterial proteins (e.g., lysozyme C, ovotransferrin, ovocalyxin-32, ovocleidin-17) constitute the molecular basis for the antimicrobial function of the cuticle^15,16^. It has been demonstrated that good eggshell cuticle quality (ESCQ) can significantly reduce the opportunity of pathogen invasion^5,17,18^, and the quantity of cuticle is a heritable trait that genetic selection to this trait can be an effective strategy to reduce transmission of microorganisms in poultry production^8,19^. Therefore, how to improve ESCQ to ensure egg biosecurity and quality and further promote poultry production has drawn a worldwide interest.

Avian birds are oviparous and produce an egg at intervals precisely controlled by the hormonal secretion and gene expression of the hypothalamo-pituitary–gonadal-oviduct axis^20,21^. The avian birds’ laying cycles are affected by factors such as breeds, age, nutrition, management, physiological status and stress, and the pause day occurs after a laying sequence as a result of cycles in ovulation/oviposition greater than 24 hours^22,23^. Previous transcriptomic and proteomic studies of hen (*Gallus gallus*) reproductive tract have well profiled the temporal and spatial transcriptome landscapes and important genes that regulate egg formation^24–30^. Cuticle deposition is also a well-defined and specific process that occurs in the uterus rather than the extension of mineralized eggshell^12^. It’s reported that there is a significant association between *ovocleidin-116*, *ovocalyxin-32*, *ovalbumin*, and estrogen receptor (*ESR1*) genes and ESCQ^8,31^. Transcriptome analysis of the uterus of hens laying eggs with good or poor cuticle quality suggested that clock genes and immediate early genes are prime candidates for the control of cuticle deposition^32^. However, specific pathways and genes that regulate cuticle deposition still remain unclear.

In the present study, transcriptomic and proteomic status of uterus from White Leghorn laying hens that produced eggs with good and poor cuticle quality (GC and PC, the top and tail of the cuticle quality distribution) were analyzed to identify unknown candidate regulatory genes involved in eggshell cuticle deposition and provide deeper insights into the biological basis of cuticle deposition. This is also an accurate study to describe the uterus transcriptome and proteome during the process of chicken eggshell cuticle deposition, providing a reference for further improving the ESCQ in laying hens.

## Results

### An overview of GC and PC hens used for transcriptomic and proteomic analysis

Earlier observations of the present study showed that the ESCQ (α value) may have great variability in the same individual. Therefore, ESCQ of eggs produced by the experimental Flock-A was monitored for the 14 consecutive laying days to obtain individuals that stably produced eggs with good or poor cuticle quality (GC or PC, the top and tail 15% of the cuticle deposition distribution) for subsequent sampling and analysis. The ESCQ was extremely better in the GC group versus the PC group as indicated by the significant difference in α value (GC 30.18 ± 7.62 vs PC 10.34 ± 3.67 α value; n = 7 and 4, respectively; F1,9 < 0.001). In other words, the GC group hens had a strong ability for cuticle deposition whilst the PC groups hens had almost no deposition of cuticle. Such difference in quantity of the cuticle deposition was quite large and suitable for this study. MST cuticle blue dye results of eggs taken from uterus of GC and PC hens during sampling demonstrated that the uterus of GC group had secreted a remarkable thicker cuticle layer than PC group (Supplementary Figure S1). This significant difference of the ESCQ phenotype was in line with expectations that the tissue samples obtained in the present study could be used for subsequent analysis.

### Analysis of uterus Transcriptomic data of GC and PC group

Seven and four biological replicate samples of uterus from GC and PC group hens during cuticle formation were analysed. A total of 590,111,516 clean reads were generated from the eleven libraries of GC and PC group (Supplementary Table S1). The reads feature summary indicated that the percentage of reads mapped to *Gallus gallus* genome was ≥ 85% (Supplementary Table S2). Differential gene expression analysis showed 53 differentially expressed genes (DEGs) (with 26 genes |log_2_ fold change|> 1) between the GC and PC group during cuticle formation. Among the 53 DEGs, there were 25 up-regulated and 28 down-regulated genes at the PC group relative to the GC group. A full list of the 53 DEGs was shown in Table 1.

**Table 1:** DEGs of PC versus GC hen uterus during cuticle deposition from the transcriptomic analysis.

Gene ID	Gene name	*padj*	log_2_FoldChange
MSTRG.9297	LOC112532778	0.000107	−3.38665
MSTRG.4468	LOC112532092	0.012042	−2.55302
MSTRG.19172	novel.522	0.006783	−2.46577
MSTRG.14500	XLOC_018691	0.031623	−2.25265
MSTRG.374243	WNT2B	0.006028	−1.51444
MSTRG.15111	LOC107054937	0.031252	−1.34327
MSTRG.6687	XLOC_008333	0.041516	−1.28973
MSTRG.1320	LOC112530330	0.047795	−1.26421
MSTRG.7221	LOC112532581	0.031623	−1.2446
MSTRG.10347	LOC107054090	0.005377	−1.1694
MSTRG.11839	LOC112533379	0.007998	−1.07028
MSTRG.492	LOC112533329	0.047795	−0.97969
MSTRG.2992	ABCB5	0.013734	−0.9499
MSTRG.12238	XLOC_015835	0.00254	−0.83726
MSTRG.15139	C1orf167	0.019222	−0.83628
MSTRG.2723	RNF32	0.031623	−0.59485
MSTRG.12443	LOC107054608	0.030626	−0.55132
MSTRG.2160	RCBTB1	0.031623	−0.52116
MSTRG.8764	LOC112532722	0.007866	−2.24E−05
MSTRG.8709	LOC112532731	0.041516	−1.71E−05
MSTRG.6076	SLC25A43	0.027852	−1.66E−05
MSTRG.2635	OBSCN	0.041516	−1.41E−05
MSTRG.4968	LOC112532084	0.005377	−1.26E−05
MSTRG.5952	LOC112532329	0.031623	−1.50E−06
MSTRG.12943	RAD9B	0.037819	−1.31E−06
MSTRG.1636	LOC112532721	0.01454	−1.24E−06
MSTRG.8539	XLOC_011557	6.01E-09	−2.64E−07
MSTRG.7568	LOC112532547	0.043812	7.44E−06
MSTRG.18849	LOC101747670	0.021437	0.394261
MSTRG.11340	PLCG2	0.009689	0.477929
MSTRG.7243	ADM	0.021437	0.660846
MSTRG.2552	TAF10	0.041516	0.667103
MSTRG.13459	PTGDS	0.006028	0.728479
MSTRG.4035	BAALC	0.031252	0.76145
MSTRG.10742	BCL2L10	0.02381	0.805203
MSTRG.430	LOC101752108	0.002939	0.866194
MSTRG.17498	PRLR	0.000107	0.90763
MSTRG.14751	ID1	0.006641	0.939962
MSTRG.16737	TNS4	0.049612	1.06834
MSTRG.16730	LOC112530344	0.00254	1.133301
MSTRG.8092	XLOC_010789	0.009494	1.161744
MSTRG.5903	FGF13	0.041516	1.167194
MSTRG.4978	LRP11	0.016463	1.19355
MSTRG.12672	LOC101749583	0.000618	1.21108
MSTRG.8621	INA	0.016688	1.484377
MSTRG.4238	LOC107050437	0.043077	1.577259
MSTRG.11542	TCTA	0.000501	1.614429
MSTRG.2443	THRSPB	0.036456	1.677864
MSTRG.2098	novel.32	0.004284	1.780021
MSTRG.11804	novel.277	0.007203	1.802508
MSTRG.4206	ARC	0.036456	1.943186
MSTRG.10115	NEU4	0.007866	2.080434
MSTRG.5983	LOC100858332	0.000273	2.450145

**Figure 1:**
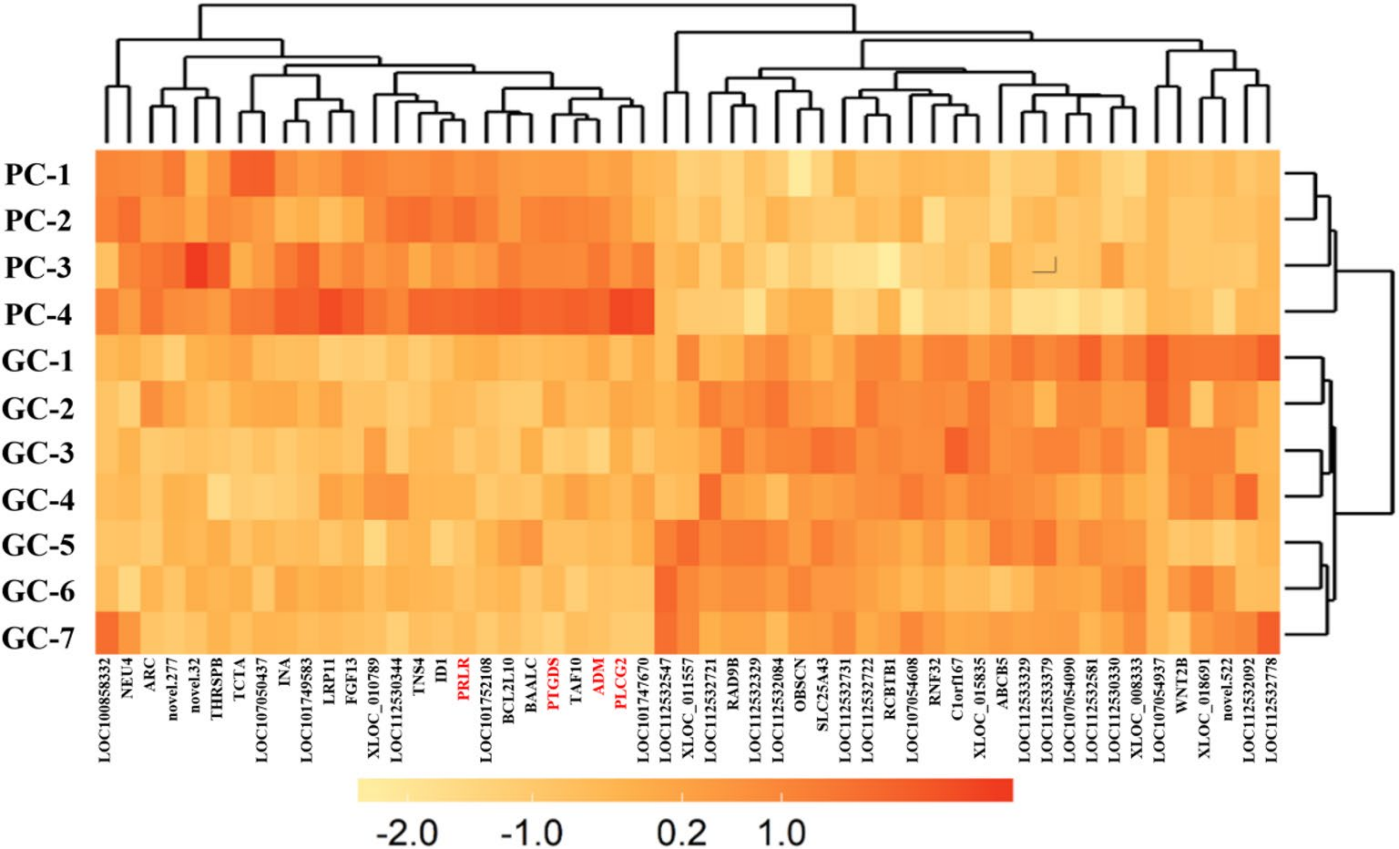
Heatmap of the DEGs between GC and PC hen uterus during cuticle deposition from the transcriptomic analysis. GC, good cuticle; PC, poor cuticle.

The heatmap of the 53 DEGs between GC and PC group was plotted and the pattern of expression for the 53 DEGs was visualised in Fig. 1, showing there were significant differences in the expression patterns of the 53 DEGs between GC and PC group.

### GO functional annotation and pathway enrichment analysis of the DEGs between GC and PC group

Functional enrichment of Gene Ontology (GO) terms and Kyoto Encyclopedia of Genes and Genomes (KEGG) pathway analysis of the 53 DEGs showed that there was no significant enriched category of GO functional annotation or KEGG pathway when subject to analysis by the DAVID Functional Annotation Tool. This may be due to the small amount of DEGs as only functions of 32 genes from the DEGs are known in the DAVID database.

### Verification of gene expression differences by quantitative real-time PCR (qPCR) analysis

**Figure 2:**
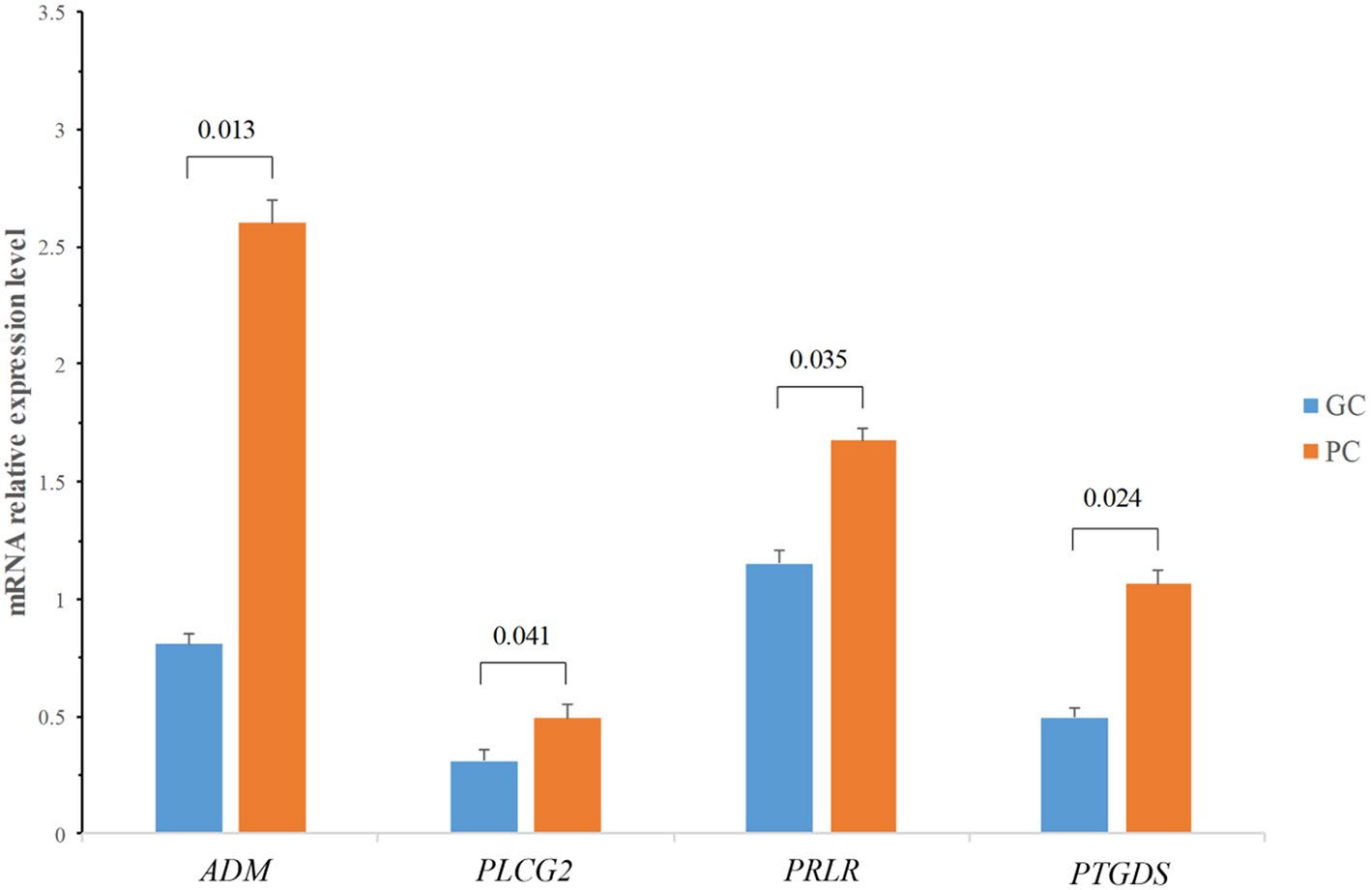
Validation of the DEGs of GC versus PC hen uterus during cuticle deposition from the transcriptomic analysis. The qPCR was performed to quantify the relative gene expression level based on the 2^−ΔΔCT^ method. For gene expression data normalization, *GAPDH* was used as a reference gene. Fold changes between the GC (good cuticle) and PC (poor cuticle) group were calculated for the genes *ADM*, *PLCG2*, *PRLR* and *PTGDS*. The Y axis shows the fold changes.

To validate the transcriptomics results, the relative expression of four genes [*ADM* (Adrenomedullin), *PLCG2* (Phospholipase C gamma 2), *PRLR* (Prolactin receptor) and *PTGDS* (Prostaglandin D2 synthase)] to *GAPDH* were determined in GC and PC group by qPCR on the basis of their known or potential influence on the hen uterus functions during cuticle deposition. The four genes were all up-regulated in PC group relative to GC group (Table 1). The qPCR results showed a highly similar expression pattern compared with the RNA-Seq analysis for the genes being validated though there was slight difference in the magnitude of changes in gene expression analyzed by RNA-Seq and qPCR (Fig. 2, Supplementary Table S3). The qPCR results suggested that the RNA-Seq data obtained in the present study provided a good reference for the study of gene expression differences in the uterus of GC and PC group during cuticle deposition.

### Proteomic analysis of uterus of GC and PC group

TMT labeling based proteomic analysis of uterus samples of GC and PC group hens (n = 4 and 3, respectively) during cuticle formation was further performed to identify differentially expressed proteins (DEPs). The peptide detection results of the TMT sequencing showed the length of the peptides is in a partial normal distribution with a mean value of 11, and the main length is between 8–13, which is reasonable for subsequent proteomic analysis. In the present study, a total of 5177 proteins were identified in the samples and 4296 proteins were quantified (Supplementary Table S4). Proteins with *P* < 0.01 by FDR correction were considered as DEPs. Among the DEGs, only PTGDS were expressed at the protein level. Further, the proteomic analysis showed no DEPs were detected between the uterus samples from the GC and PC group hens.

### Laying interval, egg quality and laying performance of GC and PC group hens

**Figure 3:**
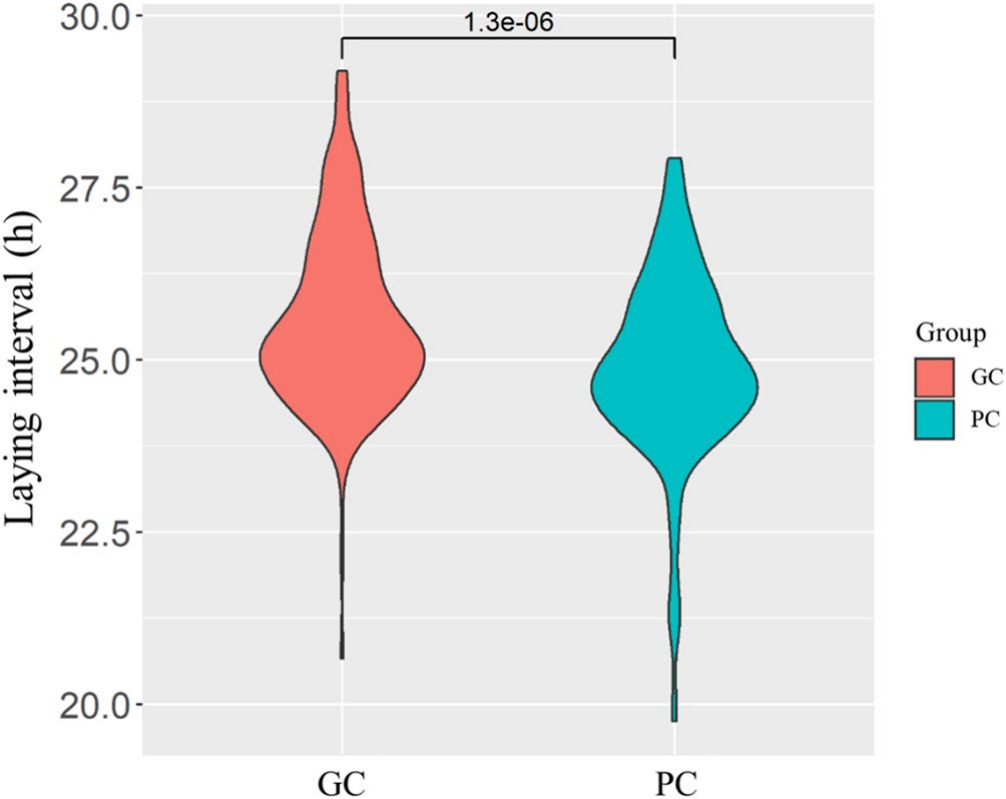
Laying interval of GC and PC group hens. The laying interval between two consecutive ovipositions was determined (at least 4 ovipositions per hen) for GC (n = 196, 60 hens with 302 ovipositions) and PC (n = 180, 60 hens with 280 ovipositions) group hens of Flock-B at 28-week old (GC 25.64 ± 1.23 vs PC 24.94 ± 1.12, h; *P* < 0.01).

**Figure 4:**
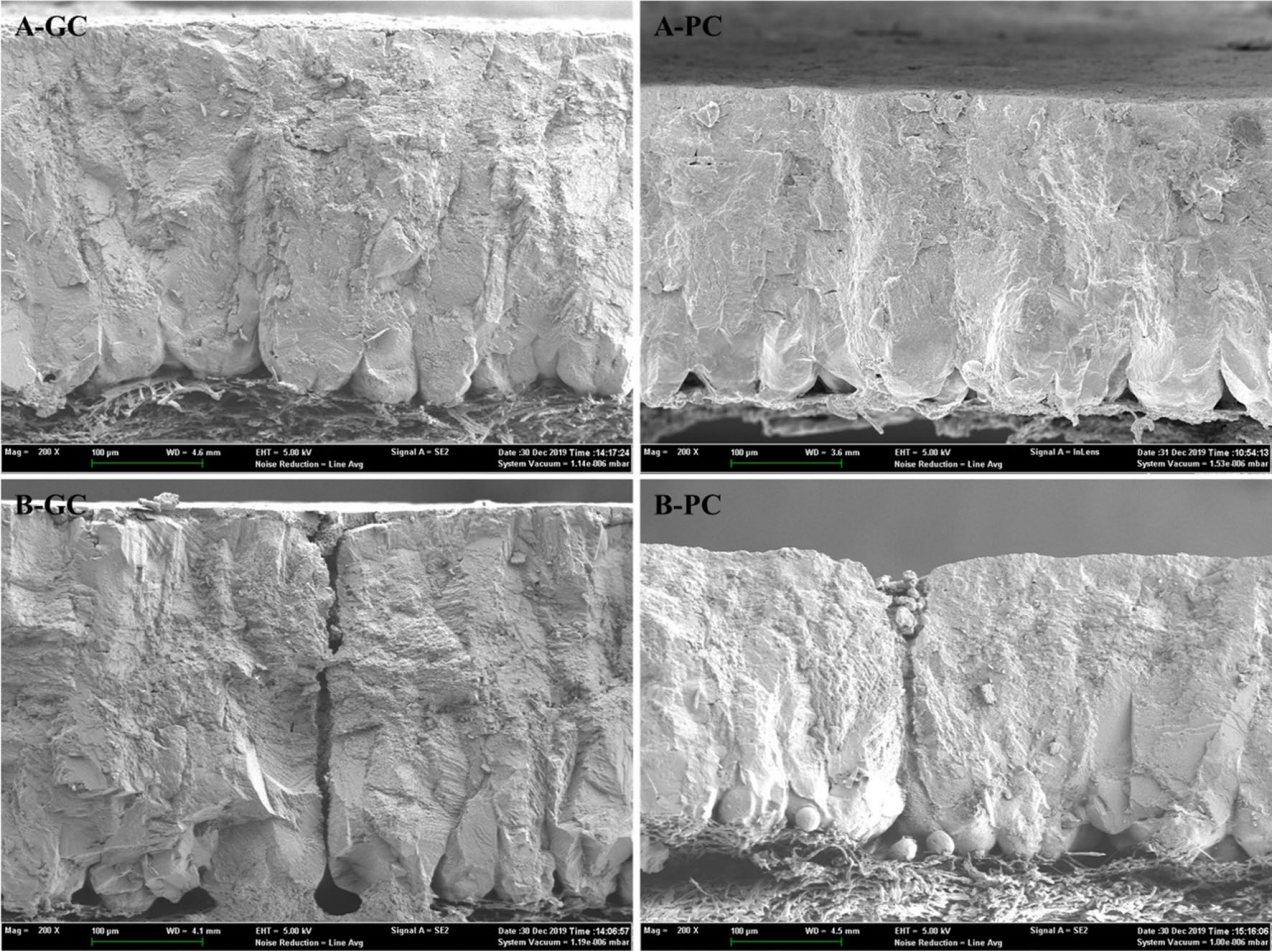
Eggshell ultrastructure of GC and PC group eggs by scanning electron microscope (SEM) (× 200). A and B, eggshell ultrastructure without and with a gas pore, respectively.

In order to further verify the results above and explore factors that affect ESCQ, laying interval, egg quality and laying performance were calculated in Flock-A and another White Leghorn laying hen flock (Flock-B) (Supplementary Table S5). Significant differences were found in the laying interval between GC and PC group in both Flock-A and -B that the GC group hens had a longer laying interval (by about 0.7 h) compared to that of PC group (*P* < 0.05) (Fig. 3; Supplementary Table S5). Further, egg quality between GC and PC group hens were measured to investigate possible reasons for the differences in laying interval between the two groups. The results of egg quality indicated that there were significant differences in eggshell thickness (EST), eggshell strength (ESS) and eggshell weight (ESW) between GC and PC group hens (*P* < 0.05), and other egg quality traits were not significantly different, suggesting the differences between GC and PC eggs were mainly reflected in the eggshell qualities (Table 2). Observations and measurements of the eggshell ultrastructure by scanning electron microscope (SEM) also visually showed the huge difference in the cuticle phenotype of GC and PC group hens (Fig. 4). Collectively, the thicker EST of GC group was largely derived from the increase in thickness of the effective layer (including the cuticle layer) (*P* < 0.05) (Fig. 4; Table 3). What’s more, Pearson’s correlation analysis also showed that the laying interval were positively correlated with the ESCQ (0.31), EST (0.32), ESS (0.33), and ESW (0.33), respectively (*P* < 0.01) (Supplementary Table S6), implying the laying interval has a considerable effect on the eggshell qualities.

**Table 2:** Egg quality between GC and PC eggs. ^1^n, 30 eggs produced by 30 different individuals in each group. ^2^α, eggshell cuticle quality (%). ^3^Eggshell thickness without eggshell membranes. ^a,b^Means within a row of the same flock that do not share a common superscript differ significantly (*P* < 0.05).

Traits	Flock-A		Flock-B	
	GC	PC	GC	PC
n^1^	30	30	30	30
α^2^	33.24 ± 6.16^a^	6.57 ± 3.00^b^	40.26 ± 5.24^a^	13.0 ± 4.03^b^
Eggshell thickness (μm)^3^	350.11 ± 27.75^a^	324.49 ± 35.44^b^	368.68 ± 23.95^a^	342.95 ± 41.28^b^
Eggshell weight (g)	5.51 ± 0.50^a^	5.10 ± 0.74^b^	5.88 ± 0.46^a^	5.53 ± 0.75^b^
Eggshell strength (kg/cm^2^)	2.87 ± 0.66^a^	2.38 ± 0.78^b^	3.65 ± 0.78^a^	3.02 ± 0.74^b^
Egg weight (g)	61.32 ± 4.46	62.32 ± 5.51	54.60 ± 3.62	55.01 ± 3.10
Egg yolk weight (g)	17.82 ± 1.47	18.43 ± 1.74	16.07 ± 3.30	16.60 ± 1.34
Egg albumen height (mm)	6.18 ± 1.14	6.03 ± 1.01	6.43 ± 1.16	6.33 ± 1.77
Egg yolk color	7.91 ± 0.79	7.85 ± 1.21	9.32 ± 0.59	9.31 ± 0.78
Haugh unit	75.67 ± 7.50	76.23 ± 11.09	81.15 ± 7.75	79.22 ± 12.30
Egg shape index	1.36 ± 0.08	1.35 ± 0.07	1.35 ± 0.08	1.35 ± 0.06

**Table 3:** Eggshell ultrastructure thickness of GC and PC eggs. ^1^n, 30 eggs produced by 30 different individuals in each group of Flock-B. ^2^α, eggshell cuticle quality (%). ^3^Effective layer thickness is the combined thickness of the palisade, vertical crystal and cuticle layer. ^a,b^Means within a row that do not share a common superscript differ significantly (*P* < 0.05).

Traits	GC	PC
n^1^	30	30
α^2^	50.55 ± 9.53^a^	0.29 ± 2.15^b^
Cuticle thickness (μm)	8.18 ± 0.83^a^	1.52 ± 0.21^b^
Effective layer thickness (μm)^3^	281.09 ± 24.57^a^	254.48 ± 32.18^b^
Mammillary layer thickness (μm)	66.04 ± 14.97	60.70 ± 14.11
Eggshell thickness (μm)	352.01 ± 23.04^a^	316.20 ± 30.58^b^

## Discussion

Egg formation in avian reproductive tract is strictly regulated by hormones and gene expression^24,33^, and the cuticle layer is the last process of egg formation in most avian species^12^. However, the genetic regulation of cuticle deposition is still poorly understood. The present study was conducted by analyzing the DEGs and DEPs of the uterus between the GC and PC group hens to elucidate potential genes and networks that regulate cuticle deposition. Besides, results of qPCR and a series of phenotypic measurements validated the RNA-Seq data. The current work not only described the differential expression profile of the uterus of GC versus PC group hens during eggshell cuticle deposition, but also revealed important genes may affecting ESCQ.

Based on the results of DEGs, multiple genes (such as *PTGDS*, *PLCG2*, *ADM* and *PRLR*) related to uterine functions and reproductive hormones were found. The qPCR results further validated the DEGs that the gene expression levels of *PTGDS*, *PLCG2, ADM* and *PRLR* in the uterus of GC group hens were all lower than those of GC group, suggesting their vital roles during cuticle formation. In mammalian studies, the interaction between these genes and their effect on uterine activity has been well shown. PTGDS is one of the prostaglandin synthases^34^, and it’s also an important catalytic enzyme for the synthesis of prostaglandins^35,36^. PLCG2, which is a type of the phospholipase C (PLC), is an important mediator of oxytocin to regulate uterine contractions^37^. Oxytocin increases the synthesis of prostaglandins by stimulating the activity of endometrial prostaglandin synthetase, and then the synergetic effect of prostaglandins and oxytocin enhances the activity of myometrium, thereby inducing parturition in mammals^38,39^. Moreover, ADM could cooperate with oxytocin and prostaglandins to participate in the rhythmical contraction and relaxation of the myometrium^40–42^. The arginine vasopressin and oxytocin are homologous nonapeptides and are known to interact with the other’s receptor with different affinities^43^. It is clear that arginine vasopressin and oxytocin arginine vasopressin play an important role in contraction of smooth muscle and parturition in mammals^44–46^. Similar to mammalian arginine vasopressin/oxytocin system, avian arginine vasotocin and mesotocin, which exert ‘oxytocic action’ inducing uterine muscle contraction during oviposition, are homologous to mammalian arginine vasopressin and oxytocin respectively^47–50^. Argine vasotocin also plays a key role in releasing prostaglandins from the uterus^51,52^. Collectively, under the influence of the hormones argine vasotocin, mesotocin and prostaglandins, uterine muscles contract leading to expulsion of the egg^52–54^. Furthermore, arginine vasotocin and prostaglandins is thought to mediate the brain to ovary signalling of oviposition timing being involved in cuticle deposition, and the premature oviposition induced by arginine vasotocin and prostaglandin significantly reduce the EST and ESCQ^12^. Previous study suggested clock gene expression in the uterus during shell formation may be responsible for controlling the cuticle deposition, and clock genes (*PER2*, *CRY2*, *CRY1*, *CLOCK* and *BMAL1*) were differentially expressed when cuticle deposition was prevented by arginine vasotocin^32^. Therefore, genes related to uterine timing mechanism and muscular events may constitute the components of cuticle deposition regulation.

Prolactin (PRL), secreted from the anterior pituitary, plays a series of roles in osmoregulation, corpus luteum formation and maintenance of broody behaviour in laying hens, and its receptor, PRLR, plays an important role in the PRL signal transduction cascade and cell growth and differentiation^55^. The *PLR* and *PRLR* genes are expressed in many tissues including the hypothalamus, ovary and oviduct^56,57^, and mediate the formation of egg quality^58^. It has been well established that the elevated plasma PRL inhibits gonadotropin release, ovum development and ovulation, resulting poor laying performance and even complete cessation of egg production in laying hens^59–61^. Previous study has also shown the inhibitory effects of excessive PRL on eggshell formation^62^. Besides, it’s clear that the elevation of PRL can significantly inhibit the cuticle deposition^12,55,63^. Therefore, the significant difference in eggshell quality (ESCQ, EST, ESS, and ESW) and egg production performance between GC and PC group hens may be related to the different expression patterns of *PRLR* gene in the uterus. However, the effects of PRL and PRLR on hen uterine function are still unclear.

Summarizing the functions of *PLCG2*, *PTGDS*, *ADM* and *PRLR* expressed in the uterus, their express patterns in the uterus regulate the muscular activities and secretion function, which may lead to the difference in egg-laying rhythm between GC and PC group hens. The relatively high expressions of *PLCG2*, *PTGDS* and *ADM* genes could increase the frequency and intensity of contraction and relaxation activities of the myometrium that might negatively affect uterine functions and eventually facilitate oviposition accompanied by a reduced duration for the egg staying in the uterus. What’s more, our results showed that the laying interval of GC group hens was significantly longer than that of PC group by about 0.7 h. The results above indicated that during the cuticle deposition period in the uterus, the frequency and intensity of myometrium contraction and relaxation in GC group hens might be lower than that of PC group hens, which created a more stable internal environment for the uterus and extend the duration of the cuticle deposition.

However, there were no DEPs between the uterus samples of GC versus PC group hens analyzed by the proteomic analysis, implying there was no significant difference in the protein composition and content to a large extent. Alternative hypothesis is that undetected DEPs may be due to the extensive posttranslational modification regulation in the biological process^64,65^. On the other hand, the extremely short half-life of the hormones may be the reason why the DEPs were not found^66–68^. The expression characteristics of the transcriptome and proteome between GC and PC group are highly similar (53 DEGs and no DEPs), suggesting that the biological processes of cuticle deposition of the two groups may be the same, but mainly the difference in the duration of cuticle deposition. However, since the duration for the uterus to secrete the cuticle is about 1–1.5 h, it was difficult to precisely obtain the uterus samples during cuticle deposition. Though we ensure that the samples and data used in this study are accurate, the relatively small sample size may not be fully representative, and the results still need to  be further verified by another study with larger sample size.

From ovulation to oviposition, it takes about 24 h for the formation of a complete egg in chicken^10,33^. A complete egg includes egg yolk, egg white, eggshell membranes, calcified eggshell and cuticle formation. The forming egg stays in different segments of the oviduct (i.e., infundibulum, magnum, isthmus and uterus) for different duration, and the egg remains in the uterus for the longest period during shell and cuticle formation, for a duration over 18 h in laying hens^10,33,69^. The egg quality measurement results showed there were no significantly differences in the egg weight, yolk weight, yolk color, albumen height, Haugh unit and egg shape index between GC and PC group. Surprisingly, the eggshell quality (ESCQ, EST, ESS and ESW) of GC group were significantly increased compared with PC group, suggesting that the longer laying interval in GC group was mainly due to the extended formation duration of the eggshell in the uterus. Furthermore, eggshell ultrastructure of GC and PC group eggs by SEM shows the significantly increased EST of GC group was largely due to the increase in the thickness of the effective layer (palisade, vertical crystal layer and cuticle), demonstrating the extended laying interval of GC group hens both positively affects the calcified shell and cuticle deposition. Moreover, the laying interval between ovipositions was positively correlated with the duration of eggshell formation, eggshell deposition rate and eggshell quality^69^, which was consistent with the Pearson’s correlation results that the laying interval and eggshell quality traits (ESCQ/EST/ESS/ESW) were positively correlated (Supplementary Table S6). Consequently, the prolonged laying interval by 0.7 h of GC group hens could explain the significant increase in the effective layer and cuticle layer thickness. Therefore, the duration of cuticle deposition in the uterus may be one of the major factors affecting ESCQ. The longer laying interval in GC group hens might be partly derived from the extended cuticle deposition time.

It was notable that the laying interval between oviposition was negatively associated with egg production traits, but it was found that GC group hens with longer laying interval have better egg production performance compared with PC group. What needs to be emphasized is that previous and present studies suggested that there was no negative genetic and phenotypic correlation between ESCQ and production traits^19^. Further, egg production performance are affected by multiple factors such as breeds, nutrition, management and the physiological state of the hen. The increase in egg production by selection largely results from the continuous ovulation of the hen with almost no pause days, which also affects laying interval between oviposition. The PC group hens had shorter mean sequence length and longer inter-sequence pause length, which might be partly associated with the expression patterns of *PLR* and *PRLR* genes as discussed above, resulting in relatively worse egg production performance. The White Leghorn laying hens used in the present study was an unselected population about 15 years, while it is remained verification whether the above results consistent with other commercial layer. In a word, though the laying interval of hens with good ESCQ was longer, there was no evidence of any adverse correlation that would prejudice the use of ESCQ as a trait for selection. The ESCQ can be effectively improved and maintained without compromising the egg production with the joint efforts of genetics, nutrition and management. An example is that Hy-Line brown, one of leading egg layer breeds, has the uniform cuticle coverage.

In summary, the physiological state of the uterus regulates the formation of eggshell ultrastructure and quality. By analyzing the transcriptome and proteome of the uterus from hens that produced eggs with good or poor cuticle quality, *PTGDS*, *PLCG2*, *ADM* and *PRLR* genes were discovered and might play crucial roles in cuticle deposition by affecting uterine secretion rhythm and function. Compared with the PC hens, the relatively low expression of these genes in the uterus ensured the eggshell and cuticle deposition duration, and lead to the good eggshell and cuticle quality of the GC hens.

## Methods

### Ethical statement

All the experimental procedures were approved by the Animal Care and Use Committee of China Agricultural University (permit number: AW08059102-1), and all the experiments and animal care protocols were performed in accordance with the Guidelines for Experimental Animals established by the committee. Animal studies were reported in compliance with the ARRIVE guidelines^70^.

### Experimental design

One 28-week-old White Leghorn laying hen flocks (n = 208, Flock-A) was used in present study for omics analysis and then another 28-week-old White Leghorn laying hen flocks (n = 574, Flock-B) were chosen for further verification tests. There was no genetic selection for the cuticle trait of the two flocks. Hens that stably produced eggs with good and poor cuticle (GC and PC, the top and tail 15% of the cuticle deposition distribution) were obtained by evaluating the cuticle quality. According to the ARRIVE guidelines^70^, in the preliminary experiment of the present study, the differences of the top and tail of the cuticle deposition distribution of the two flocks were significant large to obtain enough GC and PC hens for the experiments (GC group hens had a strong ability for cuticle deposition whilst the PC group hens had almost no secretion of cuticle). Transcriptomic, proteomic and qPCR analysis of GC and PC hen uterus tissues during cuticle deposition were designed for identifying genes involved with eggshell cuticle deposition. Subsequently, phenotypic measurements (ESCQ, laying interval, laying performance, serum hormone level, egg quality) of GC and PC group hens were carried out to further verify the results above.

### Animals and uterine tissue collection

Two hundred and eight White Leghorn hens at 28-week-old (Flock A) were selected form the National poultry Testing Center, China Agricultural University. All hens were caged (37.5 cm length, 40 cm width and 40 cm high) individually under standard conditions with a photoperiod of 16L:8D. The main ingredient of fodder is 16.5% crude protein, 2.5% crude fat, 6% fiber, 13.0% ash, 2.60–4.00% calcium, 0.60 phosphorus, 0.20–0.70% sodium chloride, 0.65% Met-Cys. The diet and water were provided ad libitum. The temperature and relative humidity of hen house was 22 ± 1 °C and 50 ± 5%, respectively.

To obtain individuals that stably produced eggs with good or poor cuticle (GC or PC) for subsequent sampling, the eggs were collected for 14 consecutive days to measure the ESCQ within 24 h after oviposition. The ESCQ evaluation was conducted according to the staining method proposed by Chen et al. previously^17^. Briefly, the ESCQ (α value) was measured based on differences in cuticle staining before and after staining with a dye solution of MST cuticle blue (MS Technologies Ltd, UK) using a spectrophotometer (CM-2600d; Konica Minolta, Japan) with the XYZ color space system. A higher α value represents to more cuticle deposition, that is better ESCQ. Each egg was measured at 3 points: the large end, equator, and small end. ESCQ per egg was determined from the mean value of these points and at least 8 eggs were collected for ESCQ measurement per hen.

Oviposition time of each hen was recorded from the eighth to fourteen day of egg collection. The oviposition time was manually recorded every 5–10 min, from 5:30 to 22:00 every day. The experimenters were trained to minimize interference with the hens. Then the oviposition time of the GC and PC group hens were estimated and the hens were euthanized by T-61 intravenously (0.4 ml/kg) 1 h before oviposition (the period for cuticle secretion) on the fifteenth day. The uterus was aseptically retracted through an abdominal incision and a small incision was then made in the centre of the uterus, and the egg in the uterus was removed for staining by MST cuticle blue within 30 min to determine whether the cuticle is deposited in the uterus during sampling. Two approximately 500 mg sample tissues was collected from the centre of the uterus and transferred directly to RNAlater (Sigma-Aldrich, Shanghai, China), quickly frozen in liquid nitrogen and stored at − 85 °C prior to total RNA extraction.

### RNA isolation, library construction, and sequencing

Total RNA was extracted from frozen uterus tissue sample using TRIzol reagent (TransGen Biotech, Beijing, China)^71^. The quality and quantity of RNA were evaluated by 1% agarose gels, NanoDrop 2000 spectrophotometer (Thermo Fisher Scientific, Wilmington, DE, USA), respectively. The RNA integrity was assessed using the RNA Nano 6000 Assay Kit of the Bioanalyzer 2100 system (Agilent Technologies, CA, USA). 3 μg RNA per sample was prepared to generate individual bar-coded sequencing libraries using NEBNext® UltraTM RNA Library Prep Kit for Illumina® (NEB, USA) following manufacturer’s recommendations. Sequencing of these libraries was performed on an Illumina HiSeq 4000 sequencing system (Illumina, San Diego, CA, USA) using the 150-bp pair-end sequencing strategy, following the manufacturer’s instructions.

### Transcriptomic data analysis

Raw data was quality controlled using the FastQC package (Babraham bioinformatics, Cambridgeshire, England). Clean reads were obtained by removing reads containing adapter, reads containing poly-N, empty reads and low quality reads from raw data. Trimmed reads were mapped on the reference chicken genome Gallus_gallus_ncbi_GCF_000002315.6_GRCg6a (https://www.ncbi.nlm.nih.gov/assembly/GCF_000002315.6) using HiSAT2.0^72,73^. The clean reads of each sample were assembled and finally merged to a transcriptome using Stringtie (http://ccb.jhu.edu/software/stringtie/) with Ensembl *Gallus gallus*.v92 as the reference^72^. Expression levels of the transcripts were quantified as RPKM (reads per kilobase per million) for gene expression analysis.

DEGs were identified using DESeq2 according to the cretiera of adjusted *P*-value < 0.05^74^. Ensembl gene IDs from each group were uploaded to the DAVID Functional Annotation Tool and analysed for gene ontology (GO) and Kyoto Encyclopaedia of Genes and Genomes (KEGG) enrichment (https://www.kegg.jp/kegg/pathway.html).^75–77^ The transcriptome data (raw mRNA-seq reads) have been deposited with the National Center for Biotechnology Information (NCBI) Sequence Read Archive (SRA) database (https://www.ncbi.nlm.nih.gov/sra/PRJNA664894).

### Sample processing and liquid chromatography coupled with tandem mass spectrometry (LC–MS/MS)

Each frozen sample (50 mg) from GC and PC group hens (n = 4 and 3, respectively) was ground in liquid nitrogen and suspended in lysis buffer consisting of 8 M urea (Sigma Aldrich, MO, USA), 10 mM dithiothreitol (DTT), and proteinase inhibitors (Merck Millipore, MA, USA). The suspension was centrifuged at 12,000×*g* for 20 min at 4 °C, and the supernatant was collected. The concentration was determined with a BCA kit (Thermo Fisher Scientific, Wilmington, DE, USA) according to the manufacturer’s protocols. After trypsin digestion, the peptides were desalted with buffer A [10 mM KH_2_PO_4_ in 25% acetonitrile (ACN), pH 3.0] and buffer B (10 mM KH_2_PO_4_, 500 mM KCl, in 25% ACN, pH 3.0) at a low rate of 1,000 μL/min, lyophilised in a centrifugal speed vacuum concentrator, reconstituted in 0.5 M TEAB and processed according to the manufacturer’s instructions for the TMT kit (Thermo Fischer Scientific, MA, USA). The tryptic peptides were separated by high-pH reverse-phase HPLC using an Agilent 300 Extend C 18 column (5 μm particle size, 4.6 mm inner diameter, 250 mm length), dissolved in 0.1% formic acid (Sigma Aldrich, MO, USA) and fractionated using EASY-Nano-LC 1000 ultra-high performance liquid phase system. Finally, fractionated peptides were exposed to an NSI source followed by tandem mass spectrometry (MS/MS) with a Q Exactive HF-X spectrometer (Thermo Fischer Scientific, MA, USA) coupled online to the UPLC system.

The MS/MS data were searched against the *Gallus gallus* database (http://www.uniprot.org/proteomes/UP000000539, chicken proteome ID: UP000000539) for peptide identification and quantification using the Maxquant search engine (v.1.6.6.0). All proteins with at least one unique peptide and false discovery rate (FDR) < 0.01 were qualified for further quantification data analysis. Protein abundance was quantified by hi-flyer through loading (SL), internal reference scaling (IRS) and trimmed mean of M values (TMM) corrections in the Maxquant search engine system. DEPs between GC and PC group were identified using limma and *P* < 0.05 was used as the cut-off for significance by FDR correction^78^.

### Quantitative real-time PCR (qPCR) analysis

Expression of mRNA was verified by qPCR with cDNA from 11 uterine tissue samples. *GAPDH* gene served as a housekeeping gene. Primer Primer (v. 5.0) was used with default parameters to design primers on exon-exon spans for selected genes (Supplementary Table S7). Total RNA was extracted from uterine tissue using TRIzol reagent as described previously. Synthesis of the cDNA was performed using a PrimeScript RT reagent kit with 1 μg of the RNA pretreated with gDNA Eraser (TaKaRa, Dalian, China) following the manufacturer’s instructions. The qPCR of the mRNA expression level of *ADM*, *PLCG2, PRLR* and *PTGDS* was performed using the Agilent Brilliant III Ultra-Fast SYBR Green qPCR Mix and MX3005P real time system (Agilent; Santa Clara, CA USA). The experiments were carried out in triplicate. The cycling conditions were 50 °C for 2 min, followed by 40 cycles at 95 °C for 15 s and 60 °C for 1 min by 42 cycles, and annealing and extension at 60 °C for 15 s. The melting curves were obtained for each sample amplified. The 2^−ΔΔCT^ method was used to quantify the relative changes in gene expression versus those of *GAPDH* from the qPCR experiments^79^, and *t*-test was used for the statistical analysis by R Language (v. 3.4.0).

### Phenotypic measurements and experimental details

After the ESCQ measurement, the eggs produced by the GC and PC hens (n = 30) of Flock-A above were randomly selected for the phenotypic measurements of egg quality traits. Egg weight, eggshell thickness (EST, mean of the large end, equator, and small end without eggshell membranes), eggshell strength (ESS), eggshell weight (ESW), yolk weight, albumen height, yolk colour, Haugh unit and egg shape index (ESI) were determined using an egg multitester (EMT-5200, robotmation, Japan), an Eggshell Strength Tester (EFG-0503, robotmation, Japan) and a digital display micrometer gauge (Mitutoyo, Kawasaki, Japan) following the manufacturer’s instructions. Laying performance (i.e., total laying days, total sequences, mean sequence length and intersequence pause length) was calculated by the 14-days egg collection. Laying interval between two consecutive ovipositions was calculated and the laying interval of each group was expressed by the average (at least 4 ovipositions per hen).

Further, Five hundred and seventy four White Leghorn hens at 28-week-old (Flock-B) were selected form the Poultry Genetic Resources and Breeding Experimental Base, College of Animal Science and Technology, China Agricultural University for subsequent verification analysis. All hens were keep in individually cage (37.5 cm length, 23.5 cm width and 40 cm high) and feeding with the same fodder and photoperiod as described above, and the diet and water were provided ad libitum. The temperature and relative humidity of hen house is 22 ± 1 °C and 50 ± 5% controlled by an automated system, respectively.

All eggs produced by Flock-B were collected, and ESCQ measurement and oviposition time recording were the same as described above for seven successive laying days. Laying recording sheet during the 27 consecutive laying days from 28-week-old age was obtained to evaluate the laying performance. The laying interval and egg quality traits of GC and PC group hens (n = 30) were measured as described above. After the egg quality measurements, eggshell pieces (about 1 cm^2^) of eggs above from the GC and PC group hens (n = 30) were taken around the equator of the egg and the pieces were mounted on an aluminum stub and gold sputter-coated using an EIKO IB-3 (EIKO Engineering CO., Ltd, Japan) for about 15 min. Thereafter, they were subjected to the SEM (JSM-7401, JEOL Ltd., Japan) for observations, photographing, and measurements of the eggshell cross-section ultrastructure. The eggshell cross-section ultrastructure in the present study refers to EST, effective layer thickness (combined thickness of the palisade, vertical crystal and cuticle layer) and mammillary layer thickness, and the determination were consistent with previous studies^31,80^.

Except for the GC and PC eggs, other eggs collected from Flock-A and Flock-B during the experiments were further measured for egg quality, respectively (at least one egg was measured per hen).

### Statistical analysis

Statistically significant differences among descriptive statistics were determined by the Student’s *t* test. The phenotypic correlation between the egg quality and laying performance traits was estimated by Pearson’s correlation coefficient. All statistical analysis and figure plotting were performed with the statistical software RStudio (v. 3.4.0). The results of basic descriptive statistics are shown in paragraphs and tables using the mean and standard deviation (mean ± SD).

## Supplementary Information

Supplementary Information 1.

Supplementary Information 2.
