# Supplementary material for: Cuticle deposition duration in the uterus is correlated with eggshell cuticle quality in White Leghorn laying hens
Source: Sci Rep. 2021 Nov 11;11:22100. doi: 10.1038/s41598-021-01718-0 (PMC8586345; doi:10.1038/s41598-021-01718-0)
Supplement: Supplementary file 1 — Supplementary Information 1. [file 41598_2021_1718_MOESM1_ESM.pdf]

Cuticle deposition duration in the uterus is correlated with eggshell cuticle quality in White Leghorn laying hens

Xia Chen[1,2†], Zhaoxiang He[1†], Xingzheng Li[1,3], Jianlou Song[1], Mingyi Huang[1], Xuefeng Shi[1], Xianyu Li[1], Junying Li[1], Guiyun Xu[1] and Jiangxia Zheng[1*]

[1]National Engineering Laboratory for Animal Breeding and MOA Key Laboratory of Animal Genetics and Breeding, College of Animal Science and Technology, China Agricultural University, Beijing, 100193, China;
[2]Institute of Animal Husbandry and Veterinary Medicine, Beijing Academy of Agriculture and Forestry Sciences, Beijing, 100097, China;
[3]Shenzhen agricultural Genome Research Institute, Chinese Academy of Agriculture Sciences, Shenzhen, 440307, China.

†These authors contributed equally to this work.

*Corresponding author: Jiangxia Zheng
Fax number: +86-010-62732741
E-mail: jxzheng@cau.edu.cn

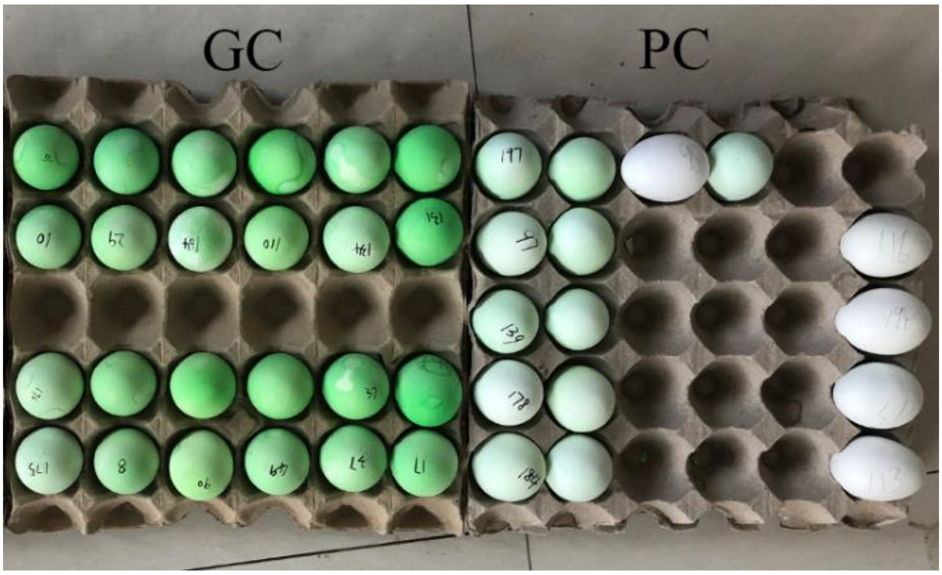

Supplementary Figure S1. MST cuticle blue dye results of eggs taken from uterus of GC and PC hens during sampling. GC, eggs with good cuticle; PC, eggs with poor cuticle. The uterus of the GC group had secreted a significant thicker cuticle layer than PC group during sampling. Individual IDs of GC group (n=7) were 10、17、29、37、49、110 and 134, respectively; individual IDs of PC group (n=4) were 97、178、184、197, respectively.

Group	Sample	Raw_Reads	Clean_Reads	Clean_Bases	Error_Rate (%)	Q20 (%)	Q30 (%)	Gc_Pct (%)
GC	G_10	44393810	43254406	6.49G	0.03	97.16	92.74	49.53
	G_17	48516666	44866314	6.73G	0.03	97.49	93.45	50.81
	G_29	49266666	46309088	6.95G	0.03	97.34	93.18	50.4
	G_37	52726666	50293592	7.54G	0.03	97.3	93.06	50.57
	G_49	62940074	58007844	8.7G	0.03	97.11	92.6	49.72
	G_110	64808242	62176528	9.33G	0.03	97.08	92.61	49.87
	G_134	54301818	52837774	7.93G	0.03	97.23	92.87	50.3
PC	P_97	58020976	57063522	8.56G	0.03	97	92.33	49.81
	P_178	64658820	63688584	9.55G	0.03	97.08	92.54	50.23
	P_184	60584332	59064708	8.86G	0.03	96.85	92.37	53.52
	P_197	53823508	52549156	7.88G	0.03	96.99	92.43	50.06

Supplementary Table S1. An overview for RNA-Seq data of uterine tissues of GC and PC group hens

Group	Sample	Total_Reads	Total_Map	Unique_Map	Multi_Map	Proper_Map	Average map
GC	G_10	43254406	39271989(90.79%)	38533677(89.09%)	738312(1.71%)	36833948(85.16%)	88.80%
	G_17	44866314	40575099(90.44%)	39776125(88.65%)	798974(1.78%)	37858366(84.38%)	
	G_29	46309088	41606842(89.85%)	40832328(88.17%)	774514(1.67%)	38720934(83.61%)	
	G_37	50293592	45464425(90.4%)	44538789(88.56%)	925636(1.84%)	42444506(84.39%)	
	G_49	58007844	52673088(90.8%)	51655283(89.05%)	1017805(1.75%)	49249010(84.9%)	
	G_110	62176528	56479776(90.84%)	55204948(88.79%)	1274828(2.05%)	52694510(84.75%)	
	G_134	52837774	48155771(91.14%)	47176118(89.28%)	979653(1.85%)	45223568(85.59%)	
PC	P_97	57063522	50688180(88.83%)	49586956(86.9%)	1101224(1.93%)	47202582(82.72%)	86.41%
	P_184	59064708	51624063(87.4%)	50213010(85.01%)	1411053(2.39%)	48068050(81.38%)	
	P_178	63688584	54904429(86.21%)	53707086(84.33%)	1197343(1.88%)	50743668(79.67%)	
	P_197	52549156	47124245(89.68%)	46199375(87.92%)	924870(1.76%)	43949086(83.63%)	

Supplementary Table S2. Sequence quality and alignment information of 11 uterine tissue samples of GC and PC group

Gene	qPCR		RNA-Seq	
	$log_2FoldChange$	*P* value	$log_2FoldChange$	*padj*
PTGDS	1.102	0.024	0.728479	0.006028
PLCG2	0.661	0.041	0.477929	0.009689
ADM	1.683	0.013	0.660846	0.021437
PRLR	0.543	0.035	0.90763	0.000107

Supplementary Table S3. Comparison of the gene expression data between RNA-Seq and qPCR.

Traits	Flock-A		Flock-B	
	GC	PC	GC	PC
n	30	30	60	60
α[1]	30.10 ± 3.62[a]	10.78 ± 1.91[b]	38.14 ± 4.93[a]	4.28 ± 1.68[b]
Lying interval (h)	24.72 ± 1.10[a]	24.06 ± 1.00[b]	25.64 ± 1.23[a]	24.94 ± 1.12[b]
Total laying days	11.83 ± 1.31[a]	10.79 ± 2.34[b]	20.67 ± 2.58[a]	18.48 ± 2.46[b]
Total sequences	1.86 ± 0.80[b]	2.36 ± 1.07[a]	6.02 ± 1.31	6.48 ± 1.45
Mean sequence length (days)	8.26 ± 4.12[a]	6.10 ± 4.00[b]	3.49 ± 1.13[a]	3.06 ± 1.05[b]
Inter-sequence pause length (days)	1.03 ± 0.86[b]	1.51 ± 0.76[a]	1.09 ± 0.26[b]	1.54 ± 0.58[a]

Supplementary Table S5. The laying performance of GC and PC group hens from Flock-A and -B. The total days used for statistics were 14 days and 27 days for Flock-A and -B, respectively. [1]α, eggshell cuticle quality (%). [a,b]Means within a row of the same flock that do not share a common superscript differ significantly ($P < 0.05$).

Traits	ESCQ	LI	EW	EST	ESS	ESW	ESW/EW	TLD	MSL	ISPL
ESCQ	1	0.26**	-0.09	0.29**	0.26**	0.23**	0.37**	0.08	-0.01	-0.08
LI	0.31**	1	/	/	/	/	/	-0.24**	-0.26**	0.15*
EW	-0.02	0.14*	1	0.13	-0.08	0.59**	-0.20	/	/	/
EST	0.25**	0.32**	0.14*	1	0.56**	0.78**	0.83**	/	/	/
ESS	0.27**	0.33**	0.08	0.59**	1	0.47**	0.65**	/	/	/
ESW	0.25**	0.33**	0.64**	0.74**	0.58**	1	0.68**	/	/	/
ESW/EW	0.34**	0.29**	-0.11	0.83**	0.67**	0.70**	1	/	/	/
TLD	0.01	-0.40**	-0.02	-0.02	-0.08	-0.04	-0.03	1	0.56**	-0.70**
MSL	-0.03	-0.50**	-0.08	-0.07	-0.11	-0.12	-0.08	0.68**	1	-0.45**
ISPL	-0.14*	0.12	0.02	-0.04	0.00	-0.06	-0.09	-0.72**	-0.32**	1

Supplementary Table S6. Pearson's correlations of the egg quality and laying performance traits. Flock-A (the upper triangles, 208 hens, 208 eggs) and Flock-B (the lower triangles, 574 hens, 832 eggs) were used for the analysis, respectively. ESCQ, eggshell cuticle quality; LI, laying interval; EW, egg weight; EST, eggshell thickness; ESS, eggshell strength; ESW, eggshell weight; TLD, total laying days; MSL, mean sequence length; ISPL, inter-sequence pause length. **P* < 0.05, ***P* < 0.01.

Gene name	Primer sequence (5′-3′)	Primer length
GAPDH	F: CTCTGTTGTTGACCTGACCT<br>R: CAACCTGGTCCTCTGTGTAT	125 bp
PRLR	F: TTTCGTGAGCCCTTGATGTGA<br>R: AATGATTGACCTGCCAGACCC	129 bp
PTGDS	F: CAAGGGTGAACAGTGCGAGAA<br>R: CACCAGGGCATACTCGTCATA	142 bp
ADM	F: CTCCTCGGGAAACTGTGC<br>R: TTCGTTTGAACTCTGTGGC	184 bp
PLCG2	F: TGAAATGGGTGGCAAGGAT<br>R: GGATACGGCAATGCTGGAC	110 bp

Supplementary Table S7. Gene names and primer sequences used in qPCR analysis
